# Chemical proteomics approach reveals the direct targets and the heme-dependent activation mechanism of artemisinin in *Plasmodium falciparum* using an artemisinin-based activity probe

**DOI:** 10.15698/mic2016.05.503

**Published:** 2016-04-05

**Authors:** Jigang Wang, Qingsong Lin

**Affiliations:** 1 The State Key Laboratory of Pharmaceutical Biotechnology, College of Life Sciences, Nanjing University, Nanjing 210023, China.; 2 Department of Biological Sciences, National University of Singapore, 117543, Singapore.

**Keywords:** targets, mechanism of action, activation, heme, artemisinin, activity-based probe, chemical proteomics, malaria, artemisinin resistance

## Abstract

Artemisinin and its analogues are currently the most effective anti-malarial drugs. The activation of artemisinin requires the cleavage of the endoperoxide bridge in the presence of iron sources. Once activated, artemisinins attack macromolecules through alkylation and propagate a series of damages, leading to parasite death. Even though several parasite proteins have been reported as artemisinin targets, the exact mechanism of action (MOA) of artemisinin is still controversial and its high potency and specificity against the malaria parasite could not be fully accounted for. Recently, we have developed an unbiased chemical proteomics approach to directly probe the MOA of artemisinin in *P. falciparum*. We synthesized an artemisinin analogue with an alkyne tag, which can be coupled with biotin through click chemistry. This enabled selective purification and identification of 124 protein targets of artemisinin. Many of these targets are critical for the parasite survival. *In vitro* assays confirmed the specific artemisinin binding and inhibition of selected targets. We thus postulated that artemisinin kills the parasite through disrupting its biochemical landscape. In addition, we showed that artemisinin activation requires heme, rather than free ferrous iron, by monitoring the extent of protein binding using a fluorescent dye coupled with the alkyne-tagged artemisinin. The extremely high level of heme released from the hemoglobin digestion by the parasite makes artemisinin exceptionally potent against late-stage parasites (trophozoite and schizont stages) compared to parasites at early ring stage, which have low level of heme, mainly derived from endogenous synthesis. Such a unique activation mechanism also confers artemisinin with extremely high specificity against the parasites, while the healthy red blood cells are unaffected. Our results provide a sound explanation of the MOA of artemisinin and its specificity against malaria parasites, which may benefit the optimization of treatment strategies and the battle against the emerging drug resistance.

The most pathogenic human malaria parasite, *Plasmodium falciparum*, is responsible for more than half-a-million deaths per year. Currently, artemisinin-based combination therapy is heavily relied to treat malaria. The artemisinin derivatives share a unique endoperoxide bridge which has been suggested to be cleaved inside *P. falciparum* by iron sources (either heme or ferrous iron) to release highly reactive carbon centered radicals. These radicals can modify and inhibit various parasite molecules, resulting in parasite death. Yet the precise MOA of artemisinin has remained obscure. Recently, our group has developed an activity-based artemisinin probe and revealed a broad spectrum of artemisinin targets that are previously unidentified. We have also provided multiple lines of evidences to demonstrate that heme, rather than free ferrous iron, is the predominant iron source for artemisinin activation. Notably, the parasite’s propagation largely relies on hemoglobin digestion, which releases high concentration of heme. Thus, such process makes artemisinin an extremely selective and potent anti-malarial drug. Once activated, the promiscuous targeting of artemisinin disrupts the essential biological processes of the parasite, leading to its death. Furthermore, we have shown that the endogenously synthesized heme might play a key role in activating artemisinin at the parasite’s early ring stage. These findings significantly enhance our understanding of artemisinin’s MOA, which may help to improve our treatment strategies against malaria, one of the most deadly diseases. The systematic identification of artemisinin targets provides invaluable information for future anti-malarial drug development. Enhancing the artemisinin activation level at the early ring stage through modulation of the parasite’s heme synthesis pathway may pave a feasible way to curb the emerging artemisinin resistance in Southeast Asia due to K13 mutations, which result in a prolonged early ring stage that diminishes the drug’s killing effect. Another possible approach to overcome the artemisinin resistance might be to extend the length of drug treatment. We anticipate that the thorough understanding of artemisinin’s MOA would help to develop better strategies to treat malaria with better treatment schemes and better drugs or drug combinations. Our list of identified artemisinin targets also provides new avenues for studying, tracking and coping with the emerging resistance of this drug.

**Figure 1:**
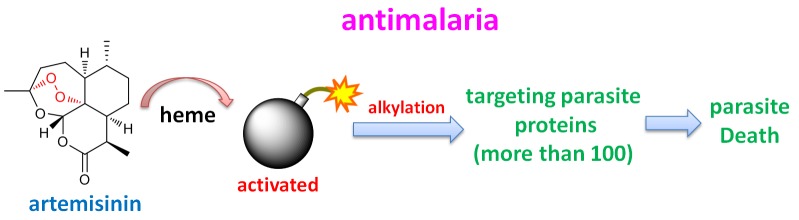
FIGURE 1: Artemisinin, activated by heme, effectively kills the malaria parasites by covalently targeting over 120 targets like a bomb. Artemisinin (chemical structure shown) can effectively kill the malaria parasites within the red blood cells (pictured). Wang *et al.* identified over 120 covalent protein targets of artemisinin. Many of them play important roles in different biological processes critical for the parasite survival. They also proved that artemisinin activation relies on heme, either biosynthesized by ring-stage parasites, or derived from hemoglobin digestion at later parasite stages.

